# Functional Interactions Between *rsks-1*/S6K, *glp-1*/Notch, and Regulators of *Caenorhabditis elegans* Fertility and Germline Stem Cell Maintenance

**DOI:** 10.1534/g3.118.200511

**Published:** 2018-08-20

**Authors:** Debasmita Roy, David J. Kahler, Chi Yun, E. Jane Albert Hubbard

**Affiliations:** * Skirball Institute of Biomolecular Medicine, Departments of Cell Biology and Pathology, New York University School of Medicine, New York, NY 10016; † NYU High Throughput Biology Laboratory, NYU Langone Health, New York, NY 10016

**Keywords:** TOR, Cyclin-E, MAPK, Translation, Cactin, RNA Exosome, Hedgehog-related

## Abstract

The proper accumulation and maintenance of stem cells is critical for organ development and homeostasis. The Notch signaling pathway maintains stem cells in diverse organisms and organ systems. In *Caenorhabditis elegans*, GLP-1/Notch activity prevents germline stem cell (GSC) differentiation. Other signaling mechanisms also influence the maintenance of GSCs, including the highly-conserved TOR substrate ribosomal protein S6 kinase (S6K). Although *C. elegans* bearing either a null mutation in *rsks-1*/S6K or a reduction-of-function (*rf*) mutation in *glp-1*/Notch produce half the normal number of adult germline progenitors, virtually all these single mutant animals are fertile. However, *glp-1(rf) rsks-1(null)* double mutant animals are all sterile, and in about half of their gonads, all GSCs differentiate, a distinctive phenotype associated with a significant reduction or loss of GLP-1 signaling. How *rsks-1*/S6K promotes GSC fate is unknown. Here, we determine that *rsks-1*/S6K acts germline-autonomously to maintain GSCs, and that it does not act through Cyclin-E or MAP kinase in this role. We found that interfering with translation also enhances *glp-1(rf)*, but that regulation through *rsks-1* cannot fully account for this effect. In a genome-scale RNAi screen for genes that act similarly to *rsks-1*/S6K, we identified 56 RNAi enhancers of *glp-1(rf)* sterility, many of which were previously not known to interact functionally with Notch. Further investigation revealed at least six candidates that, by genetic criteria, act linearly with *rsks-1*/S6K. These include genes encoding translation-related proteins, *cacn-1*/Cactin, an RNA exosome component, and a Hedgehog-related ligand. We found that additional Hedgehog-related ligands may share functional relationships with *glp-1*/Notch and *rsks-1*/S6K in maintaining germline progenitors.

Stem cells maintain tissue homeostasis throughout life. The appropriate balance between stem cell maintenance and differentiation is critical since, while too few stem cells can cause tissue degeneration, alterations in stem cell number and fate contribute to cancer. Notch is one of several evolutionarily conserved pathways that play a crucial role in regulating stem cells across different species and different organ systems, including the *C. elegans* germ line. In mammals, Notch signaling is implicated in the accumulation and/or maintenance of stem cells in diverse lineages including intestinal, muscle, and neuronal stem cells (Aster 2013; Sancho *et al.* 2015; Siebel and Lendahl 2017). Mutations that alter Notch activity are associated with many diseases, including multiple cancers (Siebel and Lendahl 2017).

p70 ribosomal protein S6 kinase (S6K) is another highly conserved signaling molecule that is best known for promoting cell growth and cell cycle progression in response to phosphorylation by Target of Rapamycin (TOR) complex 1 (TORC1). Recently, S6K has been associated with self-renewal in the context of hematopoietic stem cells (Ghosh *et al.* 2016) and neuronal regeneration (Yang *et al.* 2014) in mammals, as well as follicle stem cells in *Drosophila* (Hartman *et al.* 2013). S6K is named for its best-studied substrate ribosomal protein S6 (RPS6) (Magnuson *et al.* 2012; Meyuhas 2015). However, S6K phosphorylates many proteins, and it likely has many cellular functions including translation, proliferation, cell death, splicing, and cytoskeletal rearrangements (Fenton and Gout 2011; Magnuson *et al.* 2012). It also confers negative feedback on insulin-mediated signaling through phosphorylation of the insulin target IRS-1 (Fenton and Gout 2011; Magnuson *et al.* 2012). In mammals, S6K is encoded by two genes, S6K1 and S6K2, and regulatory interplay occurs between the two paralogs (Shima *et al.* 1998). The S6K1−/− S6K2−/− double mutant displays perinatal lethality, small size, and evidence of hyperemia, hemorrhage, as well as heart chamber dilation, but no gross anatomical defects – a surprisingly mild phenotype given the prediction that many cell-essential functions should be disrupted (Pende *et al.* 2004). In *C. elegans*, S6K is encoded by one gene, *rsks-1*, which has been implicated in growth, metabolism, lifespan regulation, germ cell development, axon regeneration, nano material toxicity, and associative learning (Long *et al.* 2002; Hansen *et al.* 2007; Pan *et al.* 2007; Sheaffer *et al.* 2008; Selman *et al.* 2009; Korta *et al.* 2012; Chen *et al.* 2013; Shi *et al.* 2013; Hubert *et al.* 2014; Zhuang *et al.* 2016; Sakai *et al.* 2017).

Previously, our lab found an unexpected functional relationship between S6K and Notch in the context of *C. elegans* germline stem cells (GSCs) (Korta *et al.* 2012). The *C. elegans* hermaphrodite germ line provides an excellent system to study stem cell accumulation and maintenance. A single somatic niche cell, the distal tip cell (DTC), expresses DSL-family ligands that activate GLP-1/Notch signaling in nearby germ cells. GLP-1/Notch activity maintains a pool of germline progenitors (that includes both GSCs and their proliferative progeny) in an undifferentiated, proliferation-competent state. As progenitors are displaced away from the distal end and escape DTC signals, they enter the meiotic pathway and eventually differentiate first into sperm and then oocytes (Hansen and Schedl 2013; Kershner *et al.* 2013). Loss of *glp-1* (or any of the core Notch signaling components) causes differentiation of all GSCs, whereas gain-of-function mutations in *glp-1* prevent differentiation and cause the formation of a germline tumor (Austin and Kimble 1987; Berry *et al.* 1997; Pepper *et al.* 2003). In contrast, the vast majority of animals bearing temperature-sensitive reduction-of-function *(rf) glp-1* mutations that are reared at a semi-permissive temperature are fertile, but they accumulate and maintain a smaller pool of GSCs. This remaining GSC pool in *glp-1(rf)* is lost completely either upon shift to the restrictive temperature (Austin and Kimble 1987) or when combined with mutations in other genes that compromise GSC maintenance (Qiao *et al.* 1995; Lee *et al.* 2007; She *et al.* 2009; Fox *et al.* 2011; Bukhari *et al.* 2012; Korta *et al.* 2012). Furthermore, average rate of cell cycle progression is unchanged (that is, it is not slower) among the germline progenitors that remain in these *glp-1(rf)* mutants at the semi-permissive temperature (Michaelson *et al.* 2010; Roy *et al.* 2016). Therefore, at the semi-permissive temperature, certain *glp-1(rf)* alleles provide a convenient sensitized genetic background to uncover extragenic regulators of GSC homeostasis.

In the context of the germ line, mutants lacking *rsks-1*/S6K act similarly to *glp-1(rf)* in the sense that they accumulate about half the number of germline progenitors and remain fertile (Korta *et al.* 2012). The reduced number of progenitors in the *rsks-1(null)* mutant is due to a combination of slower cell cycle progression and disruption of GSC maintenance that was revealed by genetic interaction with *glp-1/*Notch. Loss of *rsks-1*/S6K dramatically enhances the phenotype of a mutant with reduced *glp-1*/Notch activity: while *rsks-1(null)* and *glp-1(rf)* mutants are 100% and ∼90% fertile, respectively, all animals bearing both mutations are sterile and in roughly half of the gonads, all GSCs are lost to differentiation prior to adulthood. Here, we refer to this latter phenotype as a “loss of GSCs”. Loss of *rsks-1*/S6K also partially suppresses the penetrance of germline tumor formation in mutants with elevated *glp-1*/Notch (though in the animals where tumors do form, the tumors are smaller since cell cycle progression is slower) and can restore fertility, suggesting that *rsks-1* promotes the undifferentiated ‘GSC fate’ of the germ cells (Korta *et al.* 2012).

To further a general understanding of the functional interaction between Notch and S6K, we took advantage of experimentally tractable germline phenotypes in *C. elegans*. Our experiments revealed that *rsks-1*/S6K acts in a germline-autonomous manner, and that neither Cyclin-E nor components of MAP Kinase pathway act in a strictly linear fashion with *rsks-1* to promote GSC maintenance. We also found that while interfering with the eIF4G translation factor in *glp-1(rf)* background caused GSC maintenance defects, this effect was not exclusively dependent on *rsks*-1/S6K. We then turned to an unbiased genome-scale RNAi screening strategy to identify genes required for fertility in animals with compromised *glp-1*/Notch signaling. Our strategy targeted genes acting post-embryonically and primarily in the germ line. We found 133 genes that, when depleted by RNAi, reproducibly elevated the penetrance of sterility when combined with *glp-1(rf)*; 56 of which did not cause highly penetrant sterility in *glp-1(+)*. The majority of these 56 genes have not been previously associated with Notch signaling. We further found that 22 of these genes play a role in *C. elegans* GSC maintenance. Ultimately, using genetic criteria, we found at least 6 genes among the 22 act in a manner consistent with a genetically linear relationship with *rsks-1/*S6K to promote GSC maintenance. In addition to translation, a functional class anticipated from previous studies, our results implicate a multifunctional protein *cacn-1*/Cactin, exosome-mediated RNA processing/degradation and Hedgehog-related signaling in GSC maintenance, in concert with *rsks-1*/S6K.

## Methods

### Worm Maintenance and Strain Construction

*C. elegans* strains were derived from the Bristol N2 and maintained using standard procedures (Brenner 1974). Lab conditions included *ad libidum* feeding of OP50 *E. coli* bacteria on Nematode Growth Medium (NGM) agar plates at 20°, unless noted otherwise (Stiernagle 2006). Strains generated for this study: GC1288 *glp-1(e2141) rsks-1(sv31) III*; *naIs44 [pGC520 (pie-1p*::*rsks-1cDNA*::*GFP*::*pie-1 3′ UTR unc-119(+))]*, GC1289 *rrf-1(pk1417) I*; *glp-1(e2141) ife-1(bn127) III*, GC1326 *rrf-1(pk1417) I* ; *glp-1(e2141) rsks-1(sv31) III*, GC1329 *glp-1(e2141) rsks-1(sv31) III*; *naIs48 [pGC609 (pie-1*p**::*rsks-1 cDNA(T404A)*::*GFP*::*pie-1 3′UTR unc-119(+))]*, GC1341 *glp-1(e2141)*; *rsks-1(sv31) III*; *svIs64 [rsks-1*::*GFP]*, GC1373 *rrf-1(pk1417) I* ; *glp-1(e2141) III*; *hjSi20 [myo-2*p**::*mCherry*::*unc-54 3′UTR] IV* ; *zuIs70 [*end-1p::gfp**::*caax*; *unc-119(+)] V*, GC1374 *rrf-1(pk1417) I*; *hjSi20 [myo-2p*::*mCherry*::*unc-54 3′UTR] IV*; *zuIs70 [*end-1p::gfp**::*caax*; *unc-119(+)] V*, GC1413 *rrf-1(pk1417) I*; *naSi2(mex-5p*::*H2B*::*mCherry*::*nos-2 3′UTR) II*; *teIs113(pie-1p*::*GFP*::*H2B*::*zif-1 3′UTR) V*, GC1414 *rrf-1(pk1417) I*; *naSi2(mex-5p*::*H2B*::*mCherry*::*nos-2 3′UTR) II*; *glp-1 (e2141) III*; *teIs113(pie-1p*::*GFP*::*H2B*::*zif-1 3′UTR) V*. Allele (*naSi2*, germline mCherry::H2B) and plasmids (pGC550 used to generate *naSi2*, and pGC734 used to target *rpl-24.2* separately from C03D6.1) were also constructed for this study. For full information on these strains, alleles and plasmids, see Table S1.

### Solid media RNAi and analysis of germline progenitor zone

**Figure 1:**
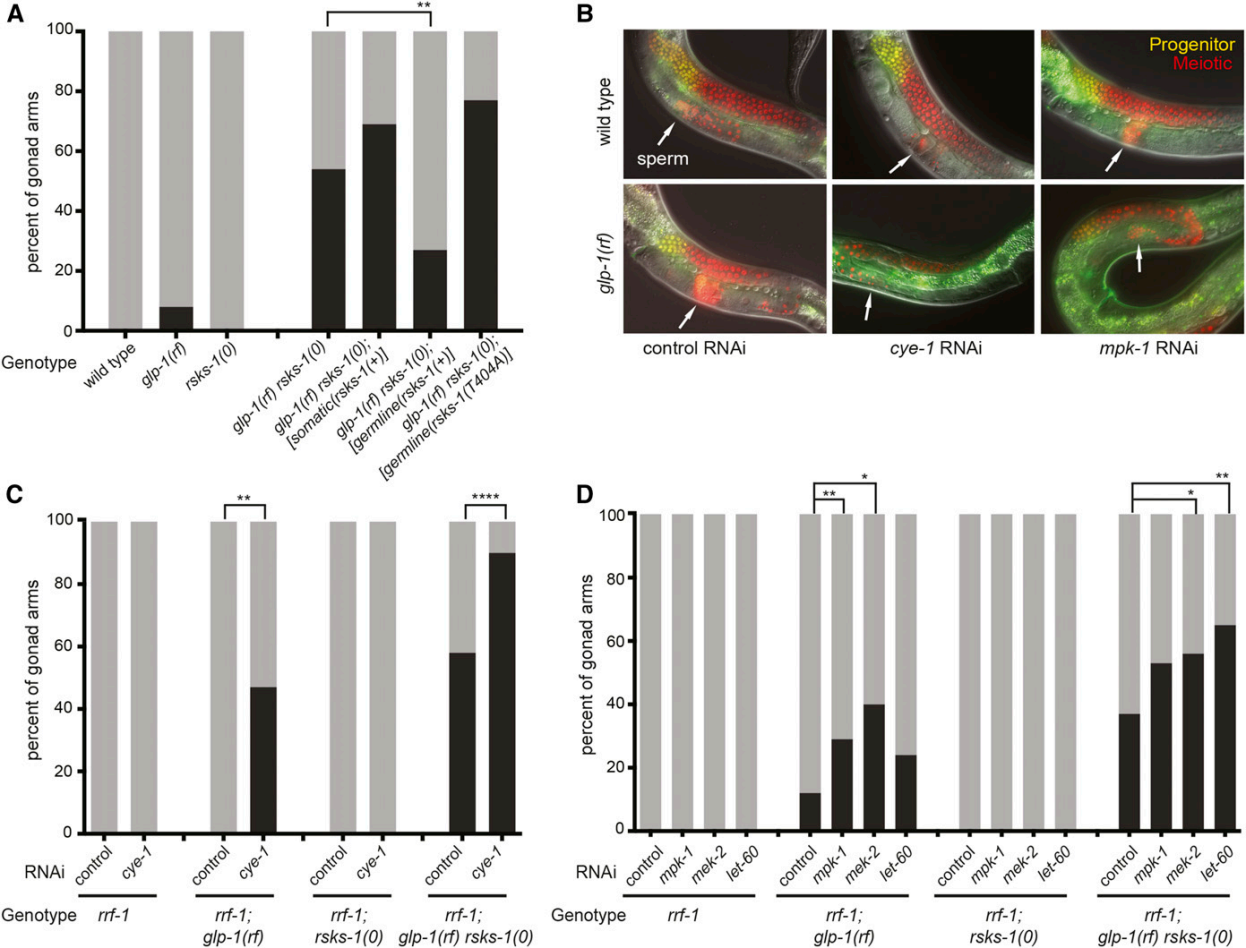
RSKS-1/S6K acts germline-autonomously and not in a simple linear pathway with Cyclin-E and MAPK to maintain GSCs when GLP-1/Notch activity is compromised. (A, C, D) Percentage of gonad arms displaying the “loss of GSCs” phenotype in which all progenitors have entered meiosis (black bars). The remainder of gonad arms maintained progenitors (gray bars). See also Figure S1 for progenitor counts. (B) Images of live animals in which mCherry labels the chromatin of germ nuclei (red; transgene insertion *naSi2*), while progenitor nuclei (yellow) are doubly marked with GFP under the control of the *pie-1* promoter and the *zif-1* 3′UTR (transgene insertion *teIs113*); see Methods for details. White arrows point to sperm. In all panels genotypes and/or genes depleted by RNAi are denoted on the X-axis; in all cases, *rrf-1* is *rrf-1(pk1417)*, *rsks-1(0)* is *rsks-1(sv31)* and *glp-1(rf)* is *glp-1(e2141)*. Statistics: 2-tailed Fisher’s exact tests, *P ≤ 0.05, **P ≤ 0.01, ****P ≤ 0.0001, see also Table S6.

**Figure 2:**
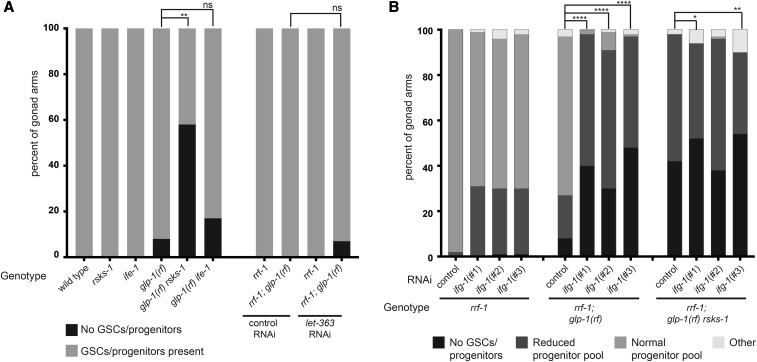
Cap-dependent translation promotes GSC maintenance. (A, B) Penetrance of GSC/progenitor defects. Panel A represents two classes of gonad arms that either show presence or absence of GSCs/progenitors. Panel B shows distribution of gonad arms across 3 categories of progenitor phenotypes: no GSCs/progenitors, a progenitor pool with a reduced number of nuclei, or a qualitatively normal progenitor pool (pattern and number of progenitors). Gonad arms were scored ‘Other’ if they displayed phenotypic abnormalities that interfered with assessment of the progenitor pool. Genotypes and genes targeted by RNAi are indicated; *rrf-1* is *rrf-1(pk1471)*, *rsks-1* is *rsks-1(sv31)*, *ife-1* is *ife-1(bn127)*, and *glp-1(rf)* is *glp-1(e2141). let-363* is *C. elegans* TOR. Clones *ifg-1(#1)*, *ifg-1(#2)*, and *ifg-1(#3)* correspond to published clones *ifg-1(C2)*, *ifg-1(C3)* and *ifg-1(N2)*, respectively, where the first two deplete both p170 and p130 isoforms of *ifg-1* and the third depletes only the p170 isoform. Statistics: 2-tailed Fisher’s exact tests for “loss of GSCs” phenotype, *P ≤ 0.05, **P ≤ 0.01, ***P ≤ 0.001, ****P ≤ 0.0001, see also Table S5.

For experiments where RNAi feeding was conducted on solid plates (Figures 1C, 1D, 2, 5B, 6, S1B, and S1C), RNAi was carried out as described (Timmons *et al.* 2001), using the empty vector L4440 in HT115 bacteria as the negative control and *cye-1* RNAi as the positive control. Animals were maintained at 15° on OP50 bacteria, and embryos collected by hypochlorite treatment (see below), and were shifted to 20° at the L1 stage when RNAi feeding commenced. Animals were scored for GSC defects at the adult molt after fixation and DAPI staining as described previously (Michaelson *et al.* 2010). Designation of the progenitor zone (Figures 1, 2, 5 and 6) and nuclei counts within the progenitor zone (Figure S1) were performed as described previously (Korta *et al.* 2012). For Figures 1, 2, 5 and 6, individual gonad arms were binned into the appropriate classes based on visual inspection. Statistical analyses: penetrance of the GSCs/progenitors present *vs.* absent was analyzed using a 2-tailed Fisher’s Exact test, and progenitor zone nuclei counts were analyzed using Welch’s *t*-test.

### Primary RNAi Screen

The primary screen was performed in a liquid-based high-throughput semi-automated manner in 96-well format (Figure S2) similar to that used by Lehner *et al.* (Lehner *et al.* 2006). We assayed 15,744 Ahringer library (Kamath *et al.* 2003) RNAi clones (∼1000 bacterial clones from the original 16,757 clones in the library were not recovered from frozen stocks), representing ∼81% of the genome. Most of the liquid handling was performed using the Matrix WellMate (ThermoScientific Cat. No. 201-20001) equipped with the Microplate Stacker (ThermoScientific Cat. No. 501-30006), and calibrated for both small and large bore 8-channel tubing.

#### Day 1:

RNAi clones from the Ahringer library (Kamath *et al.* 2003) that were maintained at -80° were replica-plated (using a 96-pin microplate replicator: Boekel Scientific Cat. No. 140500) onto LB agar plates supplemented with Ampicillin (50μg/ml) and Tetracycline (50ug/ml) in 96-well format and grown overnight at 37°.

#### Day 2:

Bacteria and worms were prepared simultaneously. Gravid worms were incubated in buffered hypochlorite solution (12ml M9 buffer [3g KH_2_PO_4_, 6g Na_2_HPO_4_, 5g NaCl, 1mL 1M MgSO_4_, H_2_0 to 1 liter]; 2ml Bleach; 1ml 5N NaOH) for 5-7min, with intermittent vortexing, to release embryos. Embryos were washed 3x in M9 buffer and collected by centrifugation at ∼3k rpm for 2min. The embryos were allowed to hatch overnight in M9 at ∼500 eggs/mL concentration. Allowing the embryos to hatch in the absence of food results in arrest at the first larval stage (L1) and thus generating a collection of synchronized L1 animals on Day 3. In parallel, on Day 2, bacteria were inoculated from the agar plates to LB liquid medium supplemented with Ampicillin (50μg/ml) in 96-deepwell plates (Fisher AB-0787) using the replicator pin. These plates were then sealed with AirPore Tape sheets (Qiagen Cat. No. 19571) to allow for exchange of air and incubated overnight (up to 16hrs) in a 37° air shaker. Positive and negative controls were manually added to empty wells on individual plates on a plate-by-plate basis. L4440 and *cye-1* were prioritized as negative and positive controls of enhancement of sterility, respectively. RNAi clones for *mek-2* and *mpk-1* were added as additional positive controls; however we found that they were variable. Additionally, we included: *dpy-5* RNAi to control for impaired somatic RNAi in *rrf-1(0)*, *lag-1* RNAi as a positive control for sterility in both *glp-1(rf)* and *glp-1(+)*, and *gfp* RNAi as a positive control for RNAi reagents and technique (*e.g.*, IPTG induction). Images from wells containing these last 3 controls: *dpy-5*, *lag-1*, and *gfp*, exhibited non-Dpy worms, sterility, and fertile GFP-negative animals, respectively.

#### Day 3:

Expression of dsRNA was induced by adding 50µl of 20mM Isopropyl β-D-1-thiogalactopyranoside (IPTG) to the 400µl overnight liquid culture (final concentration 2.2mM IPTG) and incubating for 2hrs at 37° while shaking. Following IPTG induction, bacteria cultures were centrifuged and re-suspended in S-Media (10mM Potassium Citrate; 10mM Trace metals (5mM disodium EDTA, 2.5mM FeSO_4_ •7 H_2_O, 1mM MnCl_2_•4 H_2_O, 1mM ZnSO_4_ •7 H_2_O, 0.1mM CuSO_4_ •5 H_2_O); 3mM MgSO_4_; 3mM CaCl_2_; 100μg/ml Ampicillin; 1mM IPTG; 5μg/ml Cholesterol; in S-Basal (100mM NaCl, 25mM KH_2_PO_4_, 25mM K_2_HPO_4_) using Eppendorf MixMate. L1 animals collected from the overnight hatch were re-suspended in S-Media supplemented with 0.02% Tween-20 (to minimize L1 animals adhering to the plastic) and were adjusted to a concentration of 10-15 L1/10μl by counting the number of L1 animals in 20ul of collected worms. Using an automatic Eppendorf Xplorer 12-channel repeat pipette, L1 animals in 20-30μl were combined with 40μl of bacteria culture in black-walled, clear bottom 96-well microplates (Corning Cat. No. 3904) labeled with machine-readable barcodes. Worms were incubated for 72hrs in a humidified chamber on a platform shaker at 20°.

#### Day 6:

40μl of 2mM levamisole in S-Basal was added to each well to immobilize the worms, and plates were sealed with aluminum sealing tape (Corning Cat. No. 6570). Images were acquired using Thermo Scientific ArrayScan VTI and stored as 8-bit tiff files. One 16mm^2^ field (2.5x magnification, 2x2 binning) consisting of two channels (GFP and mCherry) was acquired from the center of the well and digital images were archived for subsequent analysis. Images were exported from the HCS Studio software as jpg files to manually count the number of total and sterile worms per well based on the mCherry and GFP signals. Worms that were not visible in their entirety in the image (*e.g.*, on the edge of the well) were excluded. Total worm and sterile worm counts were uploaded to ActivityBase (IDBS) and used to calculate Z-score based on the plate, assuming that majority of the wells exhibit low/no sterility. Screening metrics were visualized using Vortex (v2014.11. Dotmatics Limited). See S3A Figure for Z-score distribution and Table S2 for raw data from the primary screen.

We also monitored worm growth since failure to reach reproductive maturity could have been scored as sterility in our assay. In cases where bacteria did not grow at all, wells contained L1 larvae after 3 days due to L1 arrest (Baugh and Sternberg 2006). In cases where worms appeared small (size of worm and proportional size of pharynx taken into consideration), suggesting that the bacteria were not sufficiently dense or other effects prevented worms from reaching adulthood in the allotted time, we noted this but did not further pursue these wells unless some of the small animals in the well also bore GFP-expressing embryos (indicating that the small size did not prevent reproductive maturity).

Because the 801 clones identified in the 1^st^ pass were candidate positives, we could no longer use Z-score as selection criteria for further analysis (see Results and Discussion). Therefore, we used a different analysis strategy for the 2^nd^ pass of the primary screen that retained a within-plate comparison to mitigate potential problems caused by plate-to-plate variability. The penetrance of sterility for each well was plotted per plate per replicate, and the point of inflection was determined as the intersection point of the two best-fit slope lines (see Figure S3B’ for an example). We selected those clones that caused a penetrance of sterility above the inflection point in at least 2 of the 3 biological replicates (366 of the 801 clones met the sterility criterion in at least 1 out of 3 replicates, and 168 met the sterility selection criterion in at least 2 out of 3 replicates) (Figure S3C).

### Bioinformatics and Statistical Analyses

Manual ‘Functional Class’ curation was performed based on WormBase (WormBase web site, http://www.wormbase.org, releases WS261-264) gene descriptions and homology information. Orthologs and disease association for specific genes were determined using the Alliance of Genome Resources web site (https://www.alliancegenome.org/), data retrieved in February 2018. Wherever possible, *C. elegans* cellular functions were prioritized over those of related genes in other species. Related candidate genes were grouped in the following categories: (1) Other: proteins with multiple functions or proteins with domain annotations and less clear cellular functions; (2) Translation: tRNA synthetases, ribosomal proteins and ribosome biogenesis factors, rRNA processing factors; (3) Signaling: components of known pathways, kinases and phosphatases; (4) Transport: ion channels, nuclear transport and vesicle functions; and (5) Unknown: genes with no obvious orthologs outside *Caenorhabditis* or no Pfam domain hits. Also see Table S3.

Statistical Overrepresentation analysis for Gene Ontology (GO) terms was performed using PANTHER v13.1. Worm Base IDs (WBGene000XXX) were entered for input and the Fisher’s Exact with FDR multiple test correction with the default settings was used to determine the highly significant and enriched GO terms. (Mi *et al.* 2013; Mi *et al.* 2017).

### Data availability statement

Strains and other reagents are available from the Caenorhabditis Genetics Center or upon request. The authors affirm that all data necessary for confirming the conclusions of the article are present within the article, figures, and table, together with supplementary Tables and Figures that have been uploaded to figshare. Figure S1 contains progenitor zone counts relevant to Figures 1 and 2. Figure S2 is a workflow diagram for the primary RNAi screen. Figure S3 shows the distribution of predicted positive wells from the screen across linkage groups, selection criteria for the 2^nd^ pass of the primary screen (including examples), and a Venn diagram of the clones selected from the 1^st^ pass. Figure S4 presents the genomic scenario and analysis for two genes (*rpl-24.2* and C03D6.1) that were targeted by a single clone from the Ahringer RNAi Library. Table S1 provides details on strains, alleles and plasmids used. Table S2 contains raw data from the primary screen. Table S3 lists the set of 133 genes, their mammalian ortholog(s) and disease associations, their distribution into the sets of 77 and 56 genes, and information on the status of the progenitor pool when each was depleted by RNAi. Table S4 lists the set of 77 genes and whether or not they were found in 7 other *C. elegans* screens. Table S5 displays in 3 tabs, the PANTHER representation analysis of the sets of 133 and 56 genes by biological process, cellular compartment, and molecular function. Table S6 shows all p- and n-values for Figures 1A, 1C, 1D, 2, 6, S1, and S4. Supplemental material available at Figshare: https://doi.org/10.25387/g3.6869828.

## Results and Discussion

### S6K acts in a germline-autonomous manner to regulate GSC fate

It was previously shown that *rsks-1/*S6K both promotes cell cycle progression (*i.e.*, promotes “proliferation”) and prevents differentiation (*i.e.*, promotes “GSC fate”), and that the combined effect of these two activities on the accumulation of germline progenitor cells is germline-autonomous (Korta *et al.* 2012). Here, we used the enhancement of the “loss of GSCs” phenotype of the reduction-of-function (*rf*) allele *glp-1(e2141)*(Priess *et al.* 1987; Dalfo *et al.* 2010) as a proxy for the effect of *rsks-1*/S6K on GSC maintenance alone, separate from cell cycle rate. At the semi-permissive temperature of 20°, ∼90% of *glp-1(rf)* animals are fertile and maintain approximately half the number of germline progenitors seen in wild type animals (the remaining ∼10% display a severe early “loss of GSCs” phenotype). However, in the double mutant with the *rsks-1*/S6K null (“*(0)*”), the penetrance of the “loss of GSCs” phenotype is ∼40–60% (Figure 1A; (Korta *et al.* 2012)) and all animals are sterile, likely due to the paucity of progenitors remaining in the gonad arms that retain some progenitors (Figure S1).

To determine whether *rsks-1*/S6K is required in the germ line to promote GSC maintenance, we re-introduced our previously characterized germline- and somatic-restricted *rsks-1*(+) transgenes (Korta *et al.* 2012) into the *glp-1(rf) rsks-1(0)* double mutant (see Methods) and assessed the percentage of gonad arms exhibiting the “loss of GSCs” phenotype. We found that germline-restricted expression of *rsks-1*(+) partially rescued the phenotype (67% retained GSCs), while somatic expression of *rsks-1*(+) did not rescue (Figure 1A). These results narrowed our focus to germline-autonomous activity of *rsks-1*/S6K for GSC maintenance.

### Neither Cyclin-E nor MAPK functionally interacts with S6K in a genetically linear manner

Similar to loss of *rsks-1*/S6K, a reduction in the activity of either Cyclin-E/CDK2 or MAP Kinase (MAPK) pathway components (*mek-2*/MAPKK, *mpk-1*/MAPK, *let-60*/Ras) enhances *glp-1(rf)* (Lee *et al.* 2007; Fox *et al.* 2011). We therefore considered the possibility that one of these might act in a linear pathway with S6K to influence GSC maintenance. We tested this idea by individually depleting *cye-1*/Cyclin-E, *mek-2*/MAPKK, *mpk-1*/MAPK, or *let-60*/Ras in the *rrf-1(0)* and *rrf-1(0)*; *glp-1(rf)* mutant backgrounds. Loss of *rrf-1* interferes with RNAi in most somatic tissues but retains full efficacy in the germ line (Sijen *et al.* 2001; Kumsta and Hansen 2012), thus reducing activity of germline-expressed genes and preventing many pleiotropic somatic phenotypes.

We reasoned that if *cye-1*/Cyclin-E were acting in a linear pathway with *rsks-1*/S6K to maintain GSCs, *cye-1* RNAi in the *rrf-1*; *glp-1(rf) rsks-1(0)* background should not further enhance the Glp-1-like “loss of GSCs” phenotype seen in *rrf-1*; *glp-1(rf) rsks-1(0)*. First, we confirmed the effects of *cye-1* RNAi feeding in the *glp-1(e2141) rf* allele (Priess *et al.* 1987; Dalfo *et al.* 2010) versus the *bn18* allele used by Fox *et al.*, and we further examined these phenotypes in live animals using a reporter for germline progenitors (Figure 1B,1C; Methods). Similar to what was previously reported (Fox *et al.* 2011), we observed that ∼45% of gonads displayed the “loss of GSCs” phenotype after *cye-1* RNAi in *rrf-1*; *glp-1(rf)*. We also confirmed that, as previously reported (Korta *et al.* 2012), ∼55% displayed the phenotype in *rrf-1*; *glp-1(rf) rsks-1(0)* with control RNAi. However, *cye-1* RNAi in *rrf-1*; *glp-1(rf) rsks-1(0)* enhanced the phenotype to 90% (Figure 1C). This additive effect on the penetrance of the “loss of GSCs” phenotype is inconsistent with a linear relationship between *cye-1*/Cyclin-E and *rsks-1*/S6K.

Results with MAPK pathway genes were also inconsistent with a solely linear role with *rsks-1*. Using the *glp-1(e2141)* allele, similar to a previous report (Lee *et al.* 2007), we observed that RNAi targeting of *mek-2*, *mpk-1*, or *let-60* in *glp-1(rf)* enhanced the “loss of GSCs” phenotype (29%, 40%, and 24%, respectively; Figure 1B,D). Similar to our observations with *cye-1* RNAi, in parallel experiments, the penetrance of the “loss of GSCs” phenotype in *glp-1(rf) rsks-1(0)* was enhanced from ∼40% with control RNAi to 53%, 56%, and 65%, when *mek-2*, *mpk-1*, or *let-60* were depleted, respectively (Figure 1D). That the enhancement is not strictly additive may indicate a minor role for an S6K-MAPK connection in GSC maintenance. However due to the variable efficacy of RNAi, it is difficult to compare. We conclude that neither Cyclin-E nor the MAPK pathway acts genetically with *rsks-1*/S6K in a solely linear manner to maintain GSCs, though the activity of the MAPK pathway may contribute to the effect of *rsks-1*/S6K.

### Reduced translation can interfere with GSC maintenance

TORC1 activity is associated with optimal translation via several mechanisms. In parallel with S6K, another well-characterized substrate of TOR is the eukaryotic Initiation Factor-4E-binding protein (4E-BP). TOR phosphorylation of 4E-BP relieves inhibition of eIF4E, thereby promoting cap-dependent translation (Gingras *et al.* 1999). Although a 4E-BP ortholog has not yet been identified by sequence analysis in *C. elegans*, our previous results support the idea that *ife-1*/eIF4E and S6K play genetically independent roles in the germ line: while mutation in either prevents normal accumulation of the germline progenitor pool, the double mutant is additive (Korta *et al.* 2012). *ife-1* encodes one of 5 *C. elegans* eIF4Es, and is the one with the strongest germline progenitor expression and function (Keiper *et al.* 2000; Henderson *et al.* 2009; Korta *et al.* 2012). Our previous experiments did not distinguish whether the roles of *ife-1/*eIF4E and *rsks-1*/S6K are similar with respect to GSC fate. Therefore, we tested whether loss of *ife-1* would behave similarly to *rsks-1* with respect to enhancement of *glp-1*/Notch, and we found that it did not (Figure 2). Despite interfering with accumulation of progenitors to a similar extent as loss of *rsks-1* (Figure S1C), loss of *ife-1* did not significantly enhance the *glp-1*/Notch “loss of GSCs” phenotype (Figure 2B). Moreover, unlike *glp-1(rf) rsks-1(0)* double mutant animals, the *glp-1(rf) ife-1(0)* double mutants were fertile, albeit with a reduced brood size. Curiously, while the *rsks-1/*S6K function in GSC maintenance depends on the conserved TOR phosphorylation site T404 (Figure 1A), as tested with the previously characterized T404A substituted transgene (Korta *et al.* 2012), reduction of *let-363*/TOR acted similarly to *ife-1* in this regard (Figures 2B, S1C) and did not enhance the “loss of GSCs” phenotype in *glp-1(rf)*. One possible explanation is that *let-363*/TOR RNAi does not fully deplete activity. Our results indicate that *let-363/*TOR RNAi was effective since the number of progenitors is significantly lower (both in *glp-1(+)* and *glp-1(rf)* backgrounds; Figure S1C). Moreover, the number of progenitors is similarly low in the *let-363/*TOR RNAi and *rsks-1(0)* alone. Therefore, if the mechanism by which the progenitor pool limitation were identical for *let-363/*TOR RNAi and *rsks-1(0)*, we might expect *let-363/*TOR RNAi to have a similarly potent effect on GSC maintenance. This expectation, based on phenotypic severity, would hold regardless of whether the *let-363/*TOR RNAi fully depletes *let-363* activity. It is also formally possible that the threshold of TOR activity required for cell cycle progression may differ from that of promoting progenitor fate and that the RNAi knock-down did not reach the level required for the latter. Full resolution of this paradox awaits further analysis.

To assess the possibility that general translation may influence GSC maintenance, we manipulated *ifg-1*/eIF4G, a component of the eIF4F translation initiation complex (Long *et al.* 2002; Rhoads *et al.* 2006). *ifg-1* provided the opportunity to partially separate the roles of cap-dependent and potential cap-independent translation in GSC maintenance. Due to alternative splicing, *ifg-1* encodes a short (p130) and a long (p170) isoform, and only the longer isoform contains the cap-binding sequence (Contreras *et al.* 2008; Contreras *et al.* 2011). Therefore, when p170 is reduced relative to p130, only cap-dependent (and not cap-independent) translation is affected. Using previously characterized RNAi reagents (Contreras *et al.* 2008) that target p170 alone (affecting cap-dependent translation) or both p170 and p130 (affecting all translation) in the *rrf-1(0)* background, we found that reduction of *ifg-1* enhances the “loss of GSCs” phenotype in *glp-1(rf)* (Figure 2A). This enhancement was observed when either p170 alone or p170 and p130 were depleted, suggesting that overall translational efficiency is important for GSC maintenance. Following the reasoning presented above for *cye-1*, we further asked whether the enhancement of the “loss of GSCs” phenotype in *glp-1(rf) rsks-1(0)* was exacerbated upon *ifg-1* RNAi relative to the control. We found a modest degree of further enhancement in the *glp-1(rf) rsks-1(0)* double mutant by *ifg-1* RNAi that targeted p170 alone (clone #1 but not #2, Figure 2A), or that targeted both p170 and p130 (clone #3, Figure 2A). We were unable to maintain a triple mutant strain bearing *ifg-1(0)*, *glp-1(rf)*, and *rsks-1(0)*, and therefore could not assess these effects with mutant analysis. Nevertheless, our results suggest enhancement of *glp-1(rf)* caused by reduced *ifg-1* activity is partially, not completely, dependent on *rsks-1*/S6K.

Our observation that *ifg-1* depletion enhances the GSC loss phenotype of *glp-1(rf)* appears to contradict our observation that loss of *ife-1* does not. While this paradox deserves further investigation, one possible explanation is that other *ife* genes, such as *ife-3*, that have a minor role in the germ line, may direct translation of specific targets key for GSC maintenance in the absence of *ife-1*.

### A genome-scale RNAi screen identifies genes required for fertility when *glp-1*/Notch is reduced

**Figure 3:**
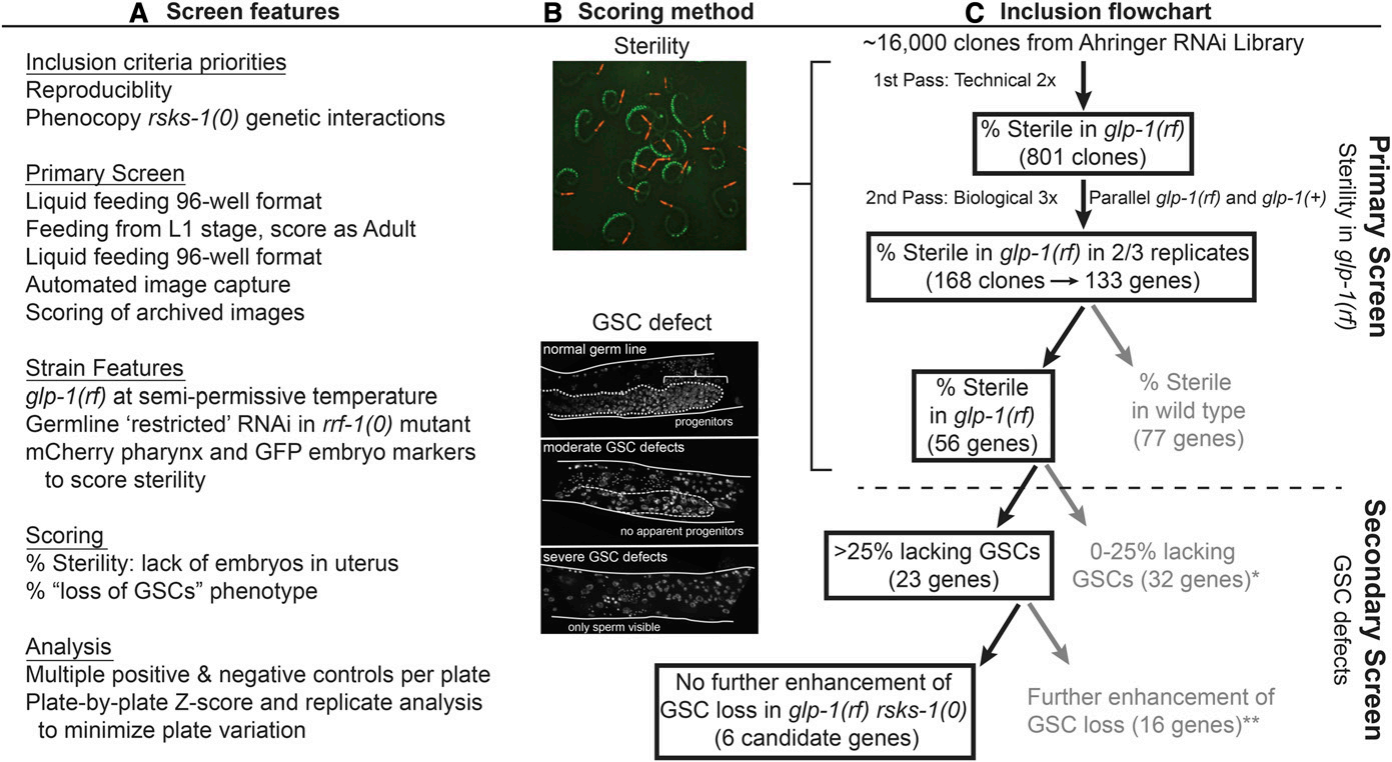
Overall RNAi screen strategy. (A) Summary of selection criteria and salient features of the screen. The starting strain for the screen was GC1373 and GC1374 was also used in the second pass (see Methods for full genotypes). (B) Representative images for scoring of sterility (top) and GSC maintenance defects (bottom). (C) Flowchart of Primary and Secondary screen strategies and results. (*) indicates exclusion of C03D6.1 from analysis (see Figure S4). (**) indicates exclusion of *rps-8* due to developmental arrest caused by RNAi (see Results).

In addition to its roles in translation, S6K influences multiple cellular processes including mRNA processing, splicing, protein folding, cell motility, and cytoskeletal rearrangements (Fenton and Gout 2011; Magnuson *et al.* 2012). To evaluate how S6K influences a Notch-mediated stem cell fate decision *in vivo*, we conducted an unbiased RNAi genetic screen and sought RNAi effects that would mimic loss of S6K. The screen used the Ahringer *C. elegans* RNAi collection that contains individual RNAi-inducing bacteria targeting ∼80% of the genes in the *C. elegans* genome (Kamath *et al.* 2003). The screen was performed in several stages (Figures 3, S2, S3 and Methods) to identify genes that when depleted, like *rsks-1(0)*, cause sterility in *glp-1(rf)* (at the semi-permissive temperature of 20°), but do not cause highly penetrant sterility in the wild type. We further screened candidates to identify those that interfere with GSC maintenance, and then identified a subset of these that did not exacerbate the penetrance of “loss of GSCs” of *glp-1(rf) rsks-1(0)* double mutants.

Five aspects of our screening and scoring strategies are notable. First, since we had determined that enhancement of *glp-1(rf)* was due to germline-autonomous activity of *rsks-1* (Figure 1A), we performed the screen in animals lacking *rrf-1*, an RNA-directed RNA polymerase that is required particularly in somatic tissues for efficient RNAi (Sijen *et al.* 2001; Kumsta and Hansen 2012). We used this strategy to focus on germline-acting genes and to avoid pleiotropic or severe somatic phenotypes. Second, we performed the RNAi feeding screen in 96-well liquid format, exposing animals to dsRNA-producing bacteria at the first larval stage (L1) to bypass embryonic lethality. We allowed these same animals to develop and scored them ∼72 hr later in the adult stage. Images captured from each well were archived and subsequently scored. Third, we adopted a very strict criterion for sterility that allowed us to assess the penetrance of sterility in a population. That is, rather than defining sterility as overall progeny production per well, we scored individual “fertile” *vs.* “sterile” animals based on the presence or absence of embryos in the uterus of each animal. We employed an *end-1p*::GFP marker (*zuIs70*; (Wehman *et al.* 2011)) to label the embryos and a *myo-2p*::mCherry pharynx marker (*hjSi20*;(Vargas *et al.* 2017)) to facilitate counting of the individual worms. Our data were recorded as “penetrance of sterility” per well. Fourth, our analysis strategy for the primary screen largely mitigated plate-to-plate and experiment-to-experiment variation. We included multiple positive and negative RNAi control clones on each plate and generated Z-scores for the individual wells on a plate-by-plate basis. We used an empirically defined Z-score cut-off of ≥1 as inclusion criteria for candidates moving forward (see Methods for further details and Figure S3). Fifth, we prioritized reproducibility using the multi-pass screening strategy outlined below.

We performed several rounds of screening. The primary screen was conducted in technical replicates (1^st^ pass) scoring for percent of animals exhibiting sterility (“penetrance of sterility”) in the *rrf-1(0)*; *glp-1(rf)* mutant with the markers described above, followed by a 2^nd^ pass where the positive candidates were re-screened for reproducibility (Figure 3). Table S2 contains raw data from the primary screen and Figure S3A shows the distribution of the Z-score values for the primary screen, 1^st^ pass results, separated by chromosome. Based on a cutoff Z-score of ≥1, (Figure S3A) we selected 801 clones from the 1^st^ pass to carry forward to the 2^nd^ pass (Figure 3).

**Figure 4:**
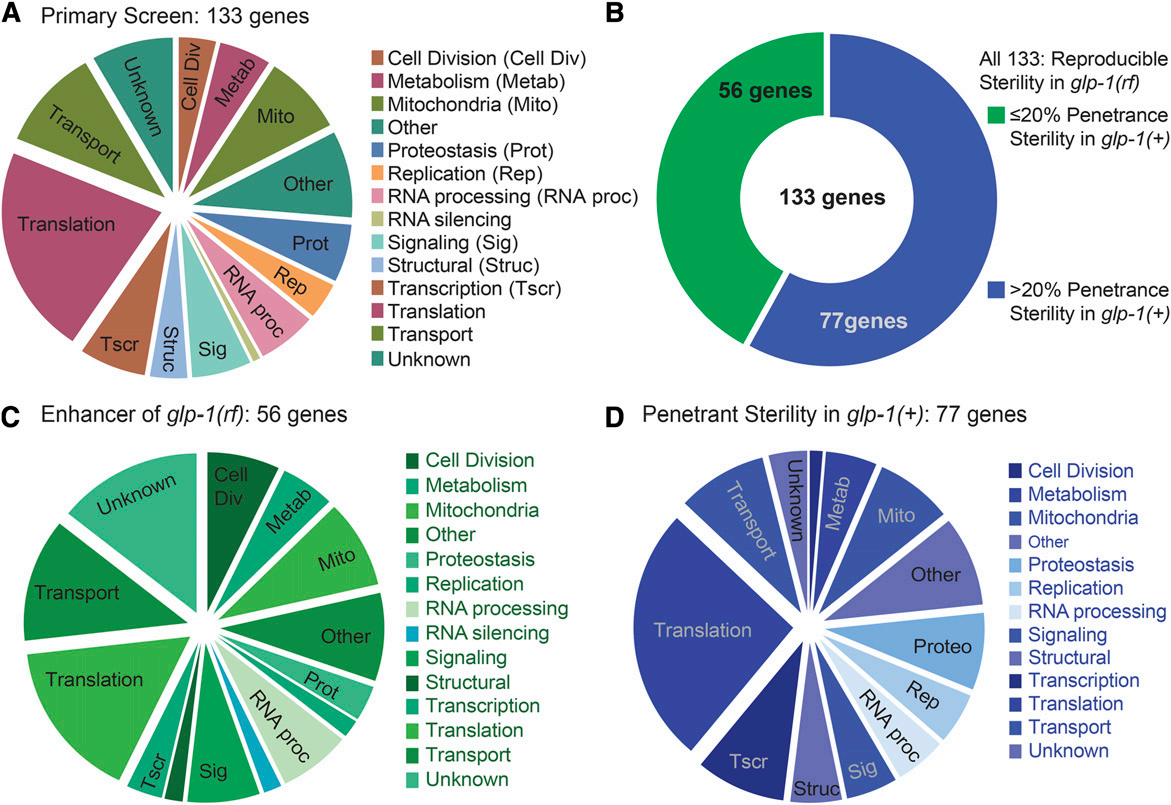
Functional classification of 133 genes identified in the Primary screen. Pie-charts summarizing the distribution of functional classes of the (A) 133 genes identified from the Primary screen. (B) The proportion of genes that reproducibly enhance sterility in *glp-1(rf)* but cause low or no sterility in *glp-1(+) vs.* those that also cause penetrant sterility in *glp-1(+)*. (C, D) The distributions of functional classes represented by the sets of 56 (C) and 77 genes (D).

In the 2^nd^ pass of the primary screen, we retested each of the 801 bacterial clones in biological triplicate in both *glp-1(rf)* (GC1373) and *glp-1(+)* (GC1374) backgrounds in parallel, and identified 168 clones that caused elevated sterility in *glp-1(rf)* in 2 of 3 replicates (Figures 3, S3C). We sequenced the inserts of the plasmids carried by bacteria in these 168 wells and identified 133 unique genes (Table S3). Using WormBase (WS257) SimpleMine and the Alliance of Genome Resources database (see Methods), we found that among the 133 genes, 112 have easily-identified mammalian orthologs, and 17 of these have clear disease associations (Table S3). Among the 133, 77 were more generally required for fertility since they displayed reproducible and penetrant (>20%) sterility in *glp-1(+)*, while the remaining 56 did not (Figure 4B). We further analyzed these two sets separately.

### Analysis of 77 genes required for penetrant fertility in *glp-1(+)*

Since our screening strategy exposed worms to RNAi only after hatching and largely limited RNAi to the germ line, we reasoned that we could potentially identify genes regulating fertility that may not have been found in screens that used maternal feeding and/or were conducted in an *rrf-1(+)* background. We therefore compared our set of 77 genes that caused marked sterility in *glp-1(+)* to those reported as “sterile (Ste)” in previous large-scale RNAi screens in *C. elegans* (Maeda *et al.* 2001; Kamath *et al.* 2003; Simmer *et al.* 2003; Rual *et al.* 2004; Fernandez *et al.* 2005; Sönnichsen *et al.* 2005). We found that 45 of our 77 genes were among the previously reported 693 unique genes (Tables S4). Using manual curation facilitated by WormBase gene descriptions and homology information (WS261), we classified these 45 common genes into 11 categories where Translation (16), Transport (6), and Proteostasis (6) were the most abundant, followed by Other, Mitochondrial, Transcription, Replication, and RNA processing categories. The Metabolism, Signaling and Structural classes were least represented (Tables S3, S4).

The remaining 32 genes represent newly-defined fertility-associated genes for which RNAi feeding in *rrf-1* mutant L1 larvae causes sterility. These genes were spread across 12 functional categories: 4 genes each in Transcription and Translation; 3 genes each in Metabolism, Mitochondrial, Other, Signaling, Structural and those with Unknown functions; 2 genes each in Replication and RNA processing; and 1 gene each in Cell Division and Transport classes (Tables S3, S4). We speculate that these were not found in previous screens due to their effects on the soma, the maternal germ line (in cases where RNAi feeding began maternally), or embryonic development.

### Analysis of the 56 genes required for optimal fertility in *glp-1(rf)*

The remaining 56 genes caused a reproducibly elevated penetrance of sterility (ranging from 20–100% penetrance) when knocked down in *rrf-1(0)*; *glp-1(rf)*, but less than 20% sterility in *rrf-1(0)*; *glp-1(+)*. These 56 genes may therefore have a more specific interaction with *glp-1*/Notch. Of these, 5 have known disease associations and 42 have evident human orthologs (Table S3). We speculate that the human orthologs of these genes may contribute to Notch-related pathologies (Siebel and Lendahl 2017).

We wondered how functional categories may differ between these 56 genes *vs.* the 77 that also caused penetrant sterility in *glp-1(+)* (Figure 4). We found that the overall categories were similarly represented, but that the distributions were not identical. For the whole set of 133 genes, we identified 14 major functional classes for which Translation was the most-abundant with 29 genes (Figure 4). A greater proportion of the 56 genes fell into Cell Division, Transport, and “Unknown”, while a greater proportion of the 77 genes fell into Translation, Transcription, and Proteostasis.

To determine the extent to which functional classes are overrepresented relative to the genome, we conducted a ‘Statistical overrepresentation test’ of Gene Ontology (GO) terms using PANTHER v13.1 (Mi *et al.* 2013; Mi *et al.* 2017)(see Methods). PANTHER recognized 131 of the 133 genes and using regulators of Biological Process (BP), expression in a specific Cellular Component (CC), and Molecular Function (MF) as the macro-classes, it classified them into 25 BP, 17 CC, and 12 MF categories. The following had the highest fold enrichment compared to the *C. elegans* reference genome: rRNA metabolic process (GO:0016072), Ribosome (GO:0005840), and Structural constituent of ribosome (GO:0003735) in the BP, CC, and MF classes, respectively (Table S5). By comparison to the set of 133, for the 56 more specific enhancers of *glp-1(rf)*, fewer categories emerged (8 BP, 12 CC, and 6 MF). Within these categories Cell proliferation (GO:0008283) and RNA localization (GO:0006403) were the most overrepresented GO terms within the BP category, while the CC and MF GO terms were similar between the sets of 133 and 56 genes.

We further compared our set of 56 genes with previously identified modifiers of Notch. We note that we did not expect to find *rsks-1* itself since a clone targeting *rsks-1* is not present in the Ahringer library. Our screen did not identify core components of the Notch signaling pathway, nor did we identify any of the 5 characterized suppressors of hypermorphic mutant of *lin-12*, the other Notch receptor homolog in *C. elegans* (Greenwald and Kovall 2013). However, a known enhancer of *glp-1(rf)*, *cye-1* (Fox *et al.* 2011), survived our filtering scheme. Although screening criteria were not identical, among the 22 previously characterized enhancers of *glp-1(rf)* (Qiao *et al.* 1995; Lamont *et al.* 2004; Tian *et al.* 2004; Lee *et al.* 2007; She *et al.* 2009; Fox *et al.* 2011; Bukhari *et al.* 2012; Dalfo *et al.* 2012; Gupta *et al.* 2015; Ames *et al.* 2017) 6 are not represented in the Ahringer library (*ego-1*, *ego-2*, *ego-3*, *ego-5*, *fbf-1*, *fbf-2*), and 3 (*daf-1*, *alg-1*, *alg-2*) act outside the germ line and therefore would not likely confer strong RNAi phenotypes in the *rrf-1* mutant . The remaining 13 (*ego-4*/*atx-2*, *csr-1*, *drh-3*, *ekl-1*, *epn-1*, *bec-1*, *atg-7*, *mrg-1*, *mpk-1*, *mek-2*, *let-60*, *cdk-2*, *lag-1*) did not meet our filtering criteria. We also compared our list with the 617 genes that have predicted or demonstrated interactions with *glp-1* as listed in WormBase (WS263), and 7 of our 56 genes overlapped (*cacn-1*, *cgh-1*, *cye-1*, *gsk-3*, *lin-39*, *prp-4*, and *teg-4*). Thus, our study adds 49 genes that functionally interact with *glp-1*/Notch.

### Comparison of our set of 56 genes with screens in other organisms

High-throughput RNAi screens have identified Notch modifiers in *Drosophila*, in cell culture or *in vivo* (Mummery-Widmer *et al.* 2009; Saj *et al.* 2010; Neumüller *et al.* 2011). Interestingly, we found orthologs of 11 of these genes in our screen (*cacn-1*, *chc-1*, *cks-1*, *eif-6*, *emb-27*, *rpl-2*, *rps-11*, *rps-8*, *teg-4*, *uaf-2*, *ubq-2*; Table 1). The majority of the common genes are either associated with translation or cell division.

**Table 1:** The set of 56 genes

Gene	Penetrant GSC defects	Functional interaction with *rsks-1*/ S6K	Mammalian ortholog(s)	*Drosophila* ortholog functional interaction with Notch	*Drosophila* or mammal ortholog involved in TORC1-S6K	*Drosophila* ortholog regulates GSCs
*atp-4*	n		ATP5J			(1)
B0280.9	n		UTP18			
B0546.5	y					
*bcas-2*	nd		BCAS2			
*bcat-1*	n		BCAT1			
C03D6.1*^a^*	y	y	Argonaute			
			PIWI family			
C27C12.3	n					
*cacn-1*	y	y	CACTIN	(2) (3)		
*cgh-1*	n		DDX6			(4)
*chc-1*	nd		CLTC	(2)		
*cks-1*	n		CKS1B	(2)		
*cyb-3*	n		CCNB3			
*cye-1*	y		CCNE		(5)	(1) (4)
*dyci-1*	y		DYNC1l1			
*eif-6*	y	y	eIF6	(2)		(1)
*emb-27*	y		CDC16	(3)	(5)	
*emb-8*	nd		POR			
*exos-3*	y	y	EXOSC3			
F16D3.6	y					
F25B4.7	y		SLC25A6			
F31F6.1	n					
F31F6.2	n					
F31F6.3	n					
F35E2.1	y					
F46C5.6	n		PPP4R4			
F53F4.11	n		RSL1D1		(6)	(4) (7)
*gsk-3*	y		GSK3A			(4) (7)
*iff-1*	n		EIF5A			
*iftb-1*	nd		EIF2S2			
K08E5.1a	n					
*lin-39*	nd		HOXA5			
*mcm-7*	n		MCM7			(4) (7)
*mma-1*	y		LRPPRC			
*mrpl-4*	y	y	MRPL4			
*npp-20*	y		SEC13			
*nxt-1*	y		NXT2			(4)
*pqn-48*	y		IFI30			
*prp-4*	n		PRPF4			(1)
*rpl-2*	n		RPL8	(2)		(1)
*rpl-24.2**^a^*	y	y	RSL24D1			
*rps-11*	n		RPS11	(3)	(5)	
*rps-23*	y		RPS23		(5)	
*rps-8*	y		RPS8	(3)	(5)	(1)
*skn-1*	n		NFE2L3			
*sop-3*	y					
*stt-3*	n		STT3B			
T12E12.1	nd		ARIH2			
*teg-4*	y		SF3B3	(2) (3)	(6)	(1)
*tsfm-1*	n		TSFM			
*twk-43*	n		KCNK18			
*uaf-2*	n		U2AF1	(2)		
*ubq-2*	nd		UBA52	(2)		(1)
*vap-1*	y		CRISP2			
*wrt-1*	y	y	DHH			
Y37A1A.3	n		SLC2A9			
Y82E9BR.3	nd		ATP5G1			

nd = not determined.

a RNAi clone overlapping with *rpl-24.2*, which is the relevant gene hit by this RNAi reagent. C03D6.1 was dropped from analysis after further investigation. See text for details.

References cited in the table:

(1) (Yan *et al.* 2014; Sanchez *et al.* 2016)

(2) (Mummery-Widmer *et al.* 2009; Saj *et al.* 2010; Neumüller *et al.* 2011)

(3) (Mummery-Widmer *et al.* 2009; Saj *et al.* 2010; Neumüller *et al.* 2011)

(4) (Yan *et al.* 2014; Sanchez *et al.* 2016)

(5) (Lindquist *et al.* 2011)

(6) (Chauvin *et al.* 2014)

(7) (Yu *et al.* 2016)

Others compared but no overlap found:

(8) (Jia *et al.* 2015; Lee *et al.* 2017b)

(9) (Jia *et al.* 2015; Lee *et al.* 2017b)

(10) (Mummery-Widmer *et al.* 2009; Saj *et al.* 2010; Neumüller *et al.* 2011)

We also wondered whether any of the genes in our set of 56 were in common with genes previously associated with TOR-S6K signaling. We compared our set to those found by Lindquist *et al.* (Lindquist *et al.* 2011) who screened for regulators of canonical TOR signaling in a *Drosophila* cell line that expressed human S6, and used phospho-RPS6 as readout, and to those found by Chauvin *et al.* (Chauvin *et al.* 2014) who compared total RNA and polysome profiles of mouse livers from wild-type *vs. S6K1^−/−^ ;S6K2^−/−^* mutants. We found 6 genes from our screen (*cye-1*, *emb-27*, *rps-11*, *rps-23*, *rps-8*, Y82E9BR.3) among orthologs to the 240 genes shown by Lindquist *et al.* (2011) to modulate TOR signaling, and 2 among 456 mRNAs identified by Chauvin *et al.* (2014) (F53F4.11 and *teg-4*). These similarities suggest that some of the other genes we found may be relevant to Notch and/or to TOR-S6K signaling in other organisms.

### Identification of genes that promote GSC maintenance in *C. elegans*

While the best-characterized role of *glp-1*/Notch in the *C. elegans* germ line is to maintain GSCs (Austin and Kimble 1987; Berry *et al.* 1997; Pepper *et al.* 2003), it also influences cytoplasmic streaming in the germ line, and oocyte growth and cellularization (Nadarajan *et al.* 2009). GLP-1 may regulate additional aspects of germline development that are experimentally inaccessible due to the severe consequences of loss of *glp-1* in the distal germ line. Indeed, our results suggest that sterility can be enhanced in *glp-1(rf)* as a result of defects other than GSC maintenance. Since our goal was to identify enhancers of *glp-1(rf)* sterility that, like *rsks-1*, act on GSCs, we further analyzed 48 of the 56 candidate genes for GSC defects (the remaining 8 were randomly excluded; see Table 1 “nd”).

**Figure 5:**
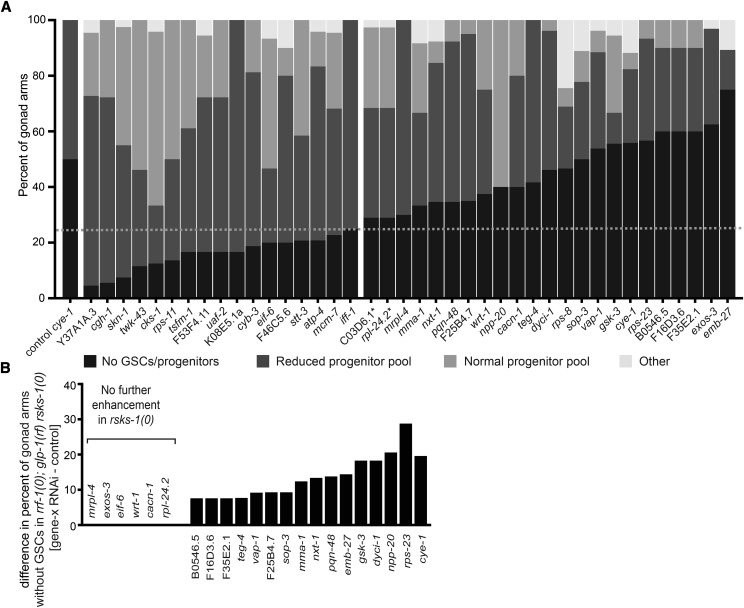
GSC maintenance defects and functional interaction with *rsks-1*/S6K. (A) Penetrance of GSC defects in *rrf-1(pk1417)*; *glp-1(e2141)* is shown as percent of gonad arms that exhibit (1) no GSCs/progenitors, (2) a reduced progenitor pool, (3) a qualitatively normal progenitor pool, or (4) display phenotypic abnormalities that precluded classification of the progenitor pool (Other). The X-axis indicates the identities of the individual genes depleted by RNAi; 10-30 gonad arms scored per experiment. The 21 genes to the right (with the exception of C03D6.1 that was shown to not influence GSC maintenance (Figure S4) and *rps-8* that showed a high proportion of gonad arms in the ‘Other’ category and could not be analyzed in the *rsks-1* mutant background), plus *eif-6* were analyzed further. *rpl-24.2 and C03D6.1 are targeted simultaneously in two independent RNAi-inducing plasmids; see text and Figure S4 for details. (B) The Y axis indicates any positive difference between the percent of gonad arms displaying the “Loss of GSCs” phenotype in *rrf-1(0)*; *glp-1(e2141) rsks-1(0)* for gene-x RNAi and control RNAi (L4440) in parallel experiments (n = 50-200 gonad arms were scored in total). RNAi targeting genes listed to the left did not exacerbate at all the loss of GSCs in *rsks-1(0) vs. rsks-1(+)*, and thereby act in a manner consistent with a linear relationship with *rsks-1*.

For this set of 48 genes, we scored for the presence or absence of GSCs as determined by DAPI staining (see Methods; Figure 3). We categorized the germline phenotypes into 4 classes: No GSCs/progenitors, Reduced progenitor pool, Normal progenitor pool (both cell number and differentiation pattern), and Other (Figure 5). We found that 40 of the 48 genes compromised GSC maintenance when depleted by RNAi, albeit at differing penetrance. Only one (*cye-1)* (Fox *et al.* 2011) was among the 22 genes previously reported to enhance of GSC defects of *glp-1(rf)* (Greenwald and Kovall 2013). In sum, our screen identified 39 genes previously unknown to functionally interact with *glp-1*/Notch in GSC maintenance.

We further analyzed 24 of the 40 genes: 23 that displayed more strongly elevated penetrance of the “loss of GSCs” phenotype (Figure 5, Table 1), plus *eif-6* that was previously found to cause a progenitor zone defect (Voutev *et al.* 2006).

We compared these 24 genes to orthologs identified in large-scale RNAi screens in *Drosophila* for genes regulating GSCs in the fly ovary (Yan *et al.* 2014; Sanchez *et al.* 2016) and testis (Yu *et al.* 2016), and in follicle stem cells (FSCs) (Jia *et al.* 2015; Lee *et al.* 2017b). We found orthologs of 6 genes (*cye-1*, *eif-6*, *gsk-3*, *mcm-7*, *nxt-1*, *rps-8*, *teg-4*) in common (none of our genes were identified in the screens for FSC regulation). *gsk-3* was the only common gene between our set and the *Drosophila* ovary and testis GSC screens. Further, orthologs of 2 of these 6 genes (*cye-1* and *rps-8*) were also identified in a screen for TORC1 signaling by Lindquist *et al.* (Lindquist *et al.* 2011) and one (*teg-4*) was found among genes that are transcriptionally responsive to S6K (Chauvin *et al.* 2014).

Within this set of 24 genes, *cacn-1* and *teg-4*, two genes implicated in splicing, caught our attention since, counter-intuitively, these genes were previously identified as enhancers of *glp-1(gain-of-function(gf))* (Mantina *et al.* 2009; Kerins *et al.* 2010). We speculate that the combination of enhancement of both *glp-1(rf)* and *glp-1(gf)* are associated with genes that are required for optimal expansion of the progenitor zone during larval stages. Sub-optimal progenitor zone expansion can reveal the activity of a “latent niche” originating in the proximal somatic gonad, which can cause enhancement of *glp-1(gf)* (Killian and Hubbard 2005; McGovern *et al.* 2009).

We also found that within this set, two genes that were initially analyzed independently in fact mapped to the same RNAi reagent. *rpl-24.2* resides inside a large intron of another gene C03D6.1, a Argonaute/PIWI family member. To distinguish whether one or both of these genes was responsible for the phenotype, we performed additional RNAi analysis with new and existing reagents to target these genes individually. We found that *rpl-24.2* RNAi caused enhanced penetrance of “loss of GSCs” in *glp-1(rf)*, but C03D6.1 RNAi did not (Figure S4). Thus, although the small RNA pathway has been implicated in germ cell fate regulation (She *et al.* 2009; Bukhari *et al.* 2012), our data indicate that C03D6.1 is not involved, and it was therefore excluded from further analysis.

Thus 23 genes went forward to the next step to be analyzed for genetic interaction with *rsks-1(0)*.

### Six candidate genes act with S6K to promote GSC maintenance

We rescreened the 23 enhancers of GSC loss in *glp-1(rf)* for their genetic interaction with *rsks-1*/S6K (see Methods). Employing the same logic described above for our analysis of *cye-1* and MAPK, we assessed the “loss of GSCs” phenotype in *glp-1(rf) rsks-1(0)* double mutant with and without *gene-x* RNAi. One candidate, *rps-8* could not be evaluated since RNAi caused developmental arrest in the *glp-1(rf) rsks-1(0)* double mutant. We found that 16 of the remaining 22 displayed a penetrance of “loss of GSCs” that exceeded the parallel control suggesting a non-linear relationship with *rsks-1* (Figure 5). We note, however that this elevated penetrance did not reach statistical significance for any of the 16, so it is possible that some of these may act linearly with *rsks-1*. We focused on the remaining 6 that did not further enhance *glp-1(rf) rsks-1(0)* whatsoever, consistent with each acting in a linear pathway with *rsks-1*/S6K (Figure 5B). These 6 candidate genes encode proteins of diverse functions. We discuss these candidates in turn below.

#### Translation related genes: eif-6, rpl-24.2, mrpl-4:

*eif-6* is the worm ortholog of the eukaryotic initiation factor-6 (eIF6), which is implicated in nucleolar assembly of the 60S ribosomal subunit and in regulation of translation and cell cycle progression in response to insulin signaling and growth factors (Basu *et al.* 2001; Gandin *et al.* 2008; Brina *et al.* 2011). *eif-6*/eIF6 also impinges on regulation of gene expression by associating with the RNA-induced silencing complex (RISC) complex. In worms, depletion of *eif-6* impedes *lin-4* miRNA mediated repression of LIN-14 and LIN-28 target proteins and mRNA, and similar effects are observed in mammalian cells (Chendrimada *et al.* 2007). It will be of interest to determine whether either of these mechanisms underlies the GSC phenotype.

*rpl-24.2* is one of two genes in *C. elegans*, *rpl-24.1* and *rpl-24.2*, that encode the large ribosomal subunit L24 protein. Depletion of either *rpl-24.1* or *rpl-24.2* from the germ line or the whole animal results in similar growth defects (Maciejowski *et al.* 2005). *mrpl-4* is an ortholog of the mitochondrial ribosomal protein L4. While ribosomal protein S6 is the best-characterized substrate of S6K (Meyuhas 2015), our results suggest that S6K may regulate – directly or indirectly – additional ribosomal subunits both cytoplasmic and mitochondrial.

#### cacn-1:

*cacn-1* is the sole *C. elegans* ortholog of Cactin, a multifunctional protein that was also found in several related screens (see above). In *C. elegans*, *cacn-1* was initially characterized in DTC migration (Tannoury *et al.* 2010). It is also required in the soma for normal oocyte development (Cecchetelli *et al.* 2016), and it interacts with the Wnt pathway to regulate *C. elegans* larval development (Labonty *et al.* 2014). In humans, Cactin was shown to negatively regulate the NFκB pathway to modulate immune response and to modulate pre-mRNA splicing and sister chromatid cohesion (Atzei *et al.* 2010; Suzuki *et al.* 2016; Zanini *et al.* 2017). Cactin is also a component of the spliceosome (Cecchetelli *et al.* 2016). How Cactin relates to S6K function remains to be determined, but S6K1 was shown to promote efficient splicing of lipogenic genes via phosphorylation of Serine-arginine protein kinase 2 (SRPK2) (Lee *et al.* 2017a) suggesting a possible link to the splicing activity of S6K.

#### exos-3:

*exos-3*, the sole *C. elegans* homolog of mammalian EXOSC-3/Rrp40, is one of the capping subunits of the conserved RNA exosome complex (Morton *et al.* 2018). In the worm, *exos-3* is an essential gene (WormBase WS264) that when inactivated by RNAi in adulthood extends lifespan and reduces fecundity (Chen *et al.* 2007), phenotypes also shared by *rsks-1* (Hansen *et al.* 2007). Together with nonsense-mediated decay genes, *exos-3* is also linked to ER homeostasis (Sakaki *et al.* 2012). Finally, *exos-3* RNAi alters the germline response to ionizing radiation by interfering with cell cycle arrest and apoptosis (Van Haaften *et al.* 2006). Our results implicate *exos-3* in GSC maintenance, together with *glp-1*/Notch and *rsks-1*/S6K.

#### “Hedgehog-related” ligand: wrt*-1*:

**Figure 6:**
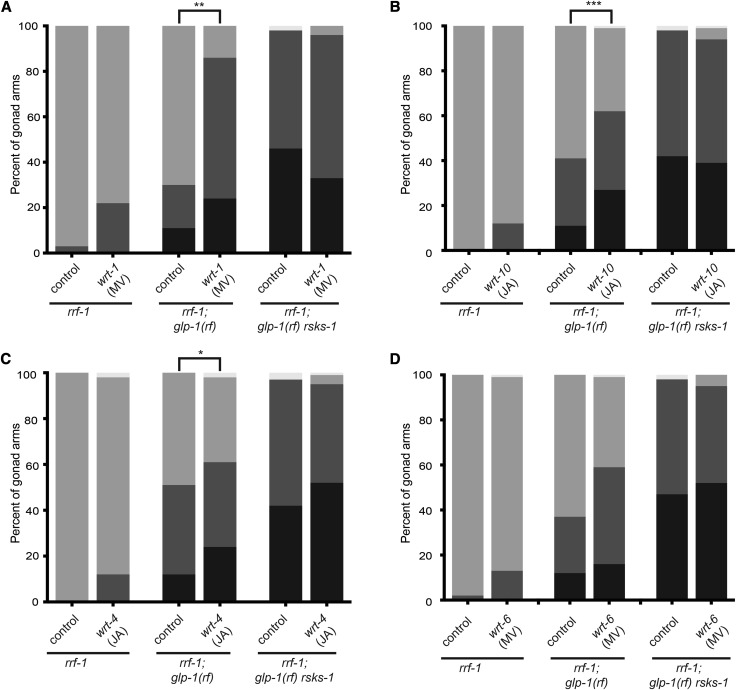
Hedgehog(Hh)-related genes *wrt-1*, *wrt-4* and *wrt-10* functionally interact with *rsks-1*/S6K to impact GSC maintenance in the *glp-1(rf)* background. (A-D) Penetrance of “loss of GSCs” phenotype is shown as percent of gonad arms (Y-axis) that have no GSCs (black). Among those that retain a progenitor pool, a reduced progenitor pool is indicated by dark gray and a qualitatively normal progenitor pool is indicated in light gray. The few remaining gonad arms displayed phenotypic abnormalities that interfered with progenitor pool assessment. *rrf-1* is *rrf-1(pk1471)*, *rsks-1* is *rsks-1(sv31)*,and *glp-1(rf)* is *glp-1(e2141)*. In all cases, *P* > 0.05 for increased penetrance of the “loss of GSCs” phenotype in *wrt-x* RNAi relative to control RNAi in the *glp-1(rf) rsks-1* double mutant. (D) Although *wrt-6* RNAi did not enhance the “loss of GSCs” phenotype in *glp-1(rf)*, it enhanced the proportion of animals with a qualitatively reduced progenitor zone (*P* < 0.0001). Statistics: 2-tailed Fisher’s exact tests for “loss of GSCs” phenotype, *P ≤ 0.05, **P ≤ 0.01, ***P ≤ 0.001, ****P ≤ 0.0001, see also Table S6.

*wrt-1* encodes a predicted secreted molecule with similarity in the C-terminal region of Hedgehog (Hh) ligands (the “Hint” or “Hog” domain), and is thus referred to as “Hedgehog (Hh)-related” (Aspock *et al.* 1999; Kuwabara *et al.* 2000; Zugasti *et al.* 2005; Bürglin and Kuwabara 2006; Bürglin 2008). While the penetrance of “loss of GSCs” in *glp-1(rf)* following *wrt-1* RNAi was modest, it was highly reproducible. We tested a second *wrt-1* RNAi reagent from the Vidal collection (Rual *et al.* 2004) that is specific for the *wrt-1* cDNA, and found that it, like the RNAi reagent from the Ahringer collection, caused a GSC maintenance defect in *glp-1(rf)* and that it did not further exacerbate the phenotype of the *glp-1(rf) rsks-1(0)* double mutant (Figure 6A). Depletion of *wrt-1* in the wild type (*rrf-1(+)*; *glp-1(+)*) did not cause any gross developmental delays or fertility defects.

The function of the Hog-domain containing proteins in *C. elegans* is poorly understood. “Hedge” domain-containing proteins originated before Eumetazoa, but the “Hint/Hog” domain likely originated even earlier, and it shares similarity with self-splicing inteins (Bürglin 2008). Not only are *C. elegans* “Hedgehog-related” ligands missing the “Hedge” domain, obvious sequence orthologs of the canonical downstream components of the Hh pathway in other systems (Smoothened, Cos2, Fu and Su(fu)) are not present (Aspock *et al.* 1999; Kuwabara *et al.* 2000; Zugasti *et al.* 2005; Bürglin and Kuwabara 2006; Bürglin 2008), suggesting divergent function relative to Hedgehog in other systems. In *C. elegans*, both the “Hh-related” and Patched gene families are greatly expanded (∼60 Hh-related genes, 3 patched orthologs (though one is likely a pseudogene), 2 dispatched orthologs and 24 patched-related genes), and the single Gli ortholog, TRA-1, is well-characterized for a role in sex-determination (Zarkower and Hodgkin 1992). The Patched ortholog *ptc-1* is required for normal germ line cytokinesis and fertility, and *ptc-3* is essential and involved in osmoregulation (Kuwabara *et al.* 2000; Soloviev *et al.* 2011). Several patched-related genes are functionally redundant and cause molting, growth and trafficking phenotypes (Zugasti *et al.* 2005), and an ancestral role has been postulated for patched-like proteins in sterol transport (Bürglin and Kuwabara 2006). Finally, RNAi targeting of some Hh-related ligands revealed similar phenotypes to the patched-related genes (growth, molting, alae formation, and trafficking defects; consistent with hypodermal expression (Aspock *et al.* 1999; Hao *et al.* 2006b), suggesting that “Hh-related” proteins and Patched may have similar rather than antagonistic roles (Zugasti *et al.* 2005). However, no previous role for Hh-related genes has been reported for GSC maintenance.

The *C. elegans* “Hh-related” proteins have been classified into 4 groups based on sequence features (Bürglin 1996; Bürglin and Kuwabara 2006; Bürglin 2008). All 10 “*warthog (wrt)*” family members contain an N terminal “Wart” domain, but they can be subdivided into two groups based on the presence (*wrt-1*, *-4*, *-6*, *-7*, *-8)* or absence (*wrt-2*, *-3*, *-5*, *-9*, *-10*) of the C-terminal Hint/SSR (Hint/ARR or “Hog”) domain (Bürglin 2008). *C. elegans* WRT-1, as expected based on the C terminal similarity to Hh ligands in other organisms, undergoes autoproteolytic cleavage (Porter *et al.* 1996). A handful of studies have investigated specific Hh-related ligands: *wrt-5* is essential, and mutants display a variety of morphological defects (Hao *et al.* 2006a); and more recently, *wrt-8* and *grl-16* were implicated in actin remodeling-dependent axon guidance, a role uncovered by their transcriptional up-regulation in *jmjd-1.2* mutants (Riveiro *et al.* 2017).

Given the large *wrt* family and the possibility of functional redundancy, we wondered whether other *wrt* family ligands may affect GSC maintenance. We tested *wrt-4*, *wrt-6*, and *wrt-10* by RNAi in *glp-1(rf)* and in the *glp-1(rf) rsks-1(0)* double mutant (Figure 6B-D). We note that although *wrt-10* is the most divergent *wrt* family member, its genomic location next to *wrt-1* suggested they may share regulatory regions. We found that like *wrt-1*, depletion of either *wrt-10* or *wrt-4*, but not *wrt-6*, significantly enhanced the “loss of GSC” phenotype in *glp-1(rf)* and that neither *wrt-10* nor *wrt-4* RNAi further exacerbated this defect in *glp-1(rf) rsks-1(0)* double mutants (Figure 6). Thus, although not easily reconciled by sequence relationships alone, at least three *C. elegans wrt*-family ligands influence GSC maintenance in a manner consistent with a linear pathway with *rsks-1*/S6K. We speculate that functional redundancy within this family may obscure its role in the germ line.

Several connections between Hh and TORC1 or S6K are emerging in other systems, but with one exception, they are not likely relevant to *C. elegans* since they are smoothened-dependent and/or converge on Gli (Filbin *et al.* 2013; D’amico *et al.* 2015; D’amico and Canettieri 2016; Miyazaki *et al.* 2016; Kim *et al.* 2017). By contrast, in the *Drosophila* ovary, S6K regulates Hh release rather than acting downstream of Hh. In the presence of dietary cholesterol, Brother of ihog (Boi) is phosphorylated in an S6K-dependent manner (Hartman *et al.* 2013). As a result, Boi tethers Hh in the absence of cholesterol, but releases it upon phosphorylation to promote follicle stem cell proliferation. While an obvious *boi* sequence ortholog is not present in the *C. elegans* genome, Boi bears similarity to adhesion proteins in *C. elegans*. Regardless, our findings provide a tractable model to explore potentially ancient roles for Hog-domain ligands, and to investigate their functional relationships with Notch and S6K.
